# Preservation of Fresh-Cut ‘Maradol’ Papaya with Polymeric Nanocapsules of Lemon Essential Oil or Curcumin

**DOI:** 10.3390/polym15173515

**Published:** 2023-08-23

**Authors:** Moises Job Galindo-Pérez, Lizbeth Martínez-Acevedo, Gustavo Vidal-Romero, Luis Eduardo Serrano-Mora, María de la Luz Zambrano-Zaragoza

**Affiliations:** 1 Departamento de Procesos y Tecnología, Universidad Autónoma Metropolitana, Unidad Cuajimalpa, Av. Vasco de Quiroga 4871, Santa Fe Cuajimalpa, Ciudad de Mexico 05348, Ciudad de Mexico, Mexico; moises.galindo@zaragoza.unam.mx; 2 Departamento del Área Farmacéutica, Facultad de Estudios Superiores Zaragoza, Universidad Nacional Autónoma de México, Campus II, Col. Ejército de Oriente, Iztapalapa, Ciudad de México 09230, Ciudad de Mexico, Mexico; gustavo.vidal@zaragoza.unam.mx; 3 Departamento de Sistemas Biológicos, Universidad Autónoma Metropolitana, Unidad Xochimilco, Calzada del Hueso 1100, Col. Villa Quietud, Coyoacán, Ciudad de Mexico 04960, Ciudad de Mexico, Mexico; liz_martinez@comunidad.unam.mx; 4 Laboratorio de Posgrado e Investigación en Tecnología Farmacéutica, Facultad de Estudios Superiores Cuautitlán, Universidad Nacional Autónoma de México, Av. 1o de Mayo s/n, Cuautitlán Izcalli 54745, Estado de Mexico, Mexico; luedserrano_h@hotmail.com; 5 Laboratorio de Procesos de Transformación de Alimentos y Tecnologías Emergentes, Departamento de Ingeniería y Tecnología, Facultad de Estudios Superiores Cuautitlán, Universidad Nacional Autónoma de México, Km 2.5 Carretera Cuautitlán–Teoloyucan, San Sebastián Xhala, Cuautitlán Izcalli 54714, Estado de Mexico, Mexico

**Keywords:** ethyl cellulose, poly-(ε-caprolactone), polymeric nanoparticles, papaya conservation, essential oils, curcumin

## Abstract

Papaya is one of the most consumed fruits in the world; however, tissue damage caused by cuts quickly leads to its decay. Therefore, this study aimed to prepare and characterize lemon oil and curcumin nanocapsules to evaluate their capacity for preserving fresh-cut papaya. Lemon essential oil and curcumin nanocapsules were prepared using ethyl cellulose (EC) and poly-(ε-caprolactone) (PCL) by the emulsification–diffusion method coupled with ultrasound. The particles had sizes smaller than 120 nm, with polydispersity indices below 0.25 and zeta potentials exceeding −12 mV, as confirmed by scanning electron microscopy. The nanoparticles remained stable for 27 days, with sedimentation being the instability mechanism observed. These nanoparticles were employed to coat fresh-cut papaya, which was stored for 17 days. The results demonstrated their remarkable efficacy in reducing the respiration rate. Furthermore, nanocapsules maintained the pH and acidity levels of the papayas for an extended period. The lemon oil/EC nanocapsule treatment retained the color better. Additionally, all systems exhibited the ability to minimize texture loss associated with reduced pectin methylesterase activity. Finally, the nanocapsules showed a notable reduction in polyphenol oxidase activity correlating with preserving total phenolic compounds in the fruit. Therefore, the lemon oil and curcumin nanoparticles formed using EC and PCL demonstrated their effectiveness in preserving fresh-cut ‘Maradol’ papaya.

## 1. Introduction

Sales of fresh-cut fruits have increased significantly in international markets, with a global annual increase of 6%. These products are ready to eat while maintaining freshness and nutritional quality [1]. Fresh-cut products are minimally processed fruits altered from their original form by peeling, slicing, dicing, cutting into strips, coring, or other similar methods, with or without washing or other treatments, before being packaged for consumer or retail use [2]. However, due to tissue cutting, a series of physicochemical and biochemical changes occur that promote a decrease in their shelf-life [3].

Papaya (*Carica papaya*) is a climacteric fruit rich in nutrients such as provitamin A, carotenoids, vitamin C, vitamin B, lycopene, dietary fiber, and minerals. It has laxative properties, reduces indigestion, and has been studied for its potential to prevent heart diseases and various types of cancer [4]. Consequently, papaya is the third most consumed tropical fruit in the world. It holds great economic and social importance, providing income for thousands of families and serving as a source of foreign exchange for producing countries. Mexico is the third-largest papaya producer globally [5]. However, fresh-cut papaya is highly perishable due to the side effects associated with tissue cutting, which accelerate respiration rate, ethylene production, and the overproduction of enzymes, ultimately leading to a decline in organoleptic and nutritional characteristics of the fruit [6]. Various alternatives have been proposed to increase the shelf-life of fresh-cut papaya, including chemical treatments, edible hydrocolloid-based coatings, and modified atmospheres, all of which have shown improvements in papaya quality [7,8,9,10]. Essential oils are secondary metabolites of plants and possess antioxidant, antimicrobial, and antifungal properties and can be incorporated into the treatments mentioned above to enhance the preservation of fresh-cut fruits [11].

Lemon essential oil is extracted from *Citrus lemon* and is a mixture of terpenes and terpenoids, with α-limonene being the main compound, accounting for approximately 60% of its composition. Lemon essential oil has demonstrated potent antimicrobial activity, inhibiting the growth of microorganisms such as *Aeromonas*, *Candida*, *Enterococcus*, *Escherichia*, and *Staphylococcus* [12,13]. It exhibits strong antioxidant properties [14]. Curcumin, the principal curcuminoid found in turmeric (*Curcuma longa*), possesses antioxidant, anti-inflammatory, antiviral, and antifungal properties. It demonstrates antioxidant activity comparable to that of vitamins C, E, and β-carotene, making turmeric a potential option for cancer prevention, liver protection, and the prevention of premature aging [15,16]. Due to its beneficial properties, curcumin has been utilized for the preservation of fresh-cut pineapple [17], pear [18], and apple [19].

Recently, nanotechnology has emerged as a promising tool for preserving fresh-cut products. Nanotechnology involves the application of material knowledge at the nanoscale and has found applications in various scientific fields, including the food processing chain. Inorganic nanoparticles such as ZnO in chitosan coatings [20] and montmorillonite embedded in a whitemouth croaker protein isolated matrix [21], oregano essential oil-based nanoemulsion [22], and citral-based nanoemulsion [23] have been used for the preservation of fresh-cut papaya.

Polymeric nanoparticles include nanocapsules and nanospheres. Nanocapsules (NCs) are vesicular structures capable of encapsulating substances with hydrophobic or hydrophilic characteristics surrounded by a polymeric barrier. Meanwhile, nanospheres are dense polymeric matrices in which the compounds are dispersed, dissolved, or chemically bound to the polymer matrix [24]. Polymeric nanoparticles have been used to encapsulate active compounds, which protect the encapsulated components from the external environment, reducing their degradation caused by heat, light, oxygen, and pH, thereby increasing their physicochemical stability [25]. In addition, polymeric nanoparticles can enable controlled release of the encapsulated components, maximizing their functionality. Materials for nanoencapsulation of active compounds are generally biodegradable synthetic polymers or FDA-approved semisynthetic or natural biopolymers for food contact [26]. Ethyl cellulose (EC) is a polymer derived from cellulose approved by the FDA as a food additive [27]. Moreover, poly-(ε-caprolactone) (PCL) is a biodegradable polyester approved by the FDA for use in drug delivery systems and widely used in food packaging [28].

Therefore, this research aimed to develop and characterize nanocapsules of lemon essential oil and curcumin using EC and PCL as biopolymer coatings for preserving fresh-cut ‘Maradol’ papaya.

## 2. Materials and Methods

### 2.1. Chemical Materials

Poly-(ε-caprolactone) (Mw ≈ 80,000), polyvinyl alcohol (PVA), lemon essential oil (*Citrus lemon*), and curcumin (*Curcuma longa*) were purchased from Sigma-Aldrich^®^ (St. Louis, MO, USA). Analytical grade ethyl acetate was acquired from Fermont^®^ (Mexico City, Mexico). Octenyl succinic anhydride starch (OSA-starch) was supplied from Makymat^®^ (Naucalpan, State of Mexico, Mexico). All other used reagents were at least analytical grade.

### 2.2. Biological Material

Papaya (*Carica papaya*) var. ‘Maradol’ fruits were obtained from a fruit distribution center in the area (Cuautitln Izcalli, State of Mexico, Mexico). They were selected according to their size and shape, with a maturity level of 5 (80–90% yellow surface) and without the presence of mechanical or microbiological damage.

### 2.3. Polymeric Nanocapsule Preparation

The nanocapsules (NCs) were prepared by the emulsification–diffusion method using ultrasonic homogenization according to the optimized conditions by Galindo-Pérez et al. (2018) [29]. Briefly, the stabilizers (PVA and OSA-starch) were dissolved in the aqueous phase at a concentration of 30 g/L. PCL or EC (307 mg) and lemon essential oil or curcumin (237 mg) were dissolved in the organic phase (water saturated ethyl acetate). Subsequently, both solutions were emulsified with an ultrasonic homogenizer at a frequency of 26 kHz (UP200HT; Helshier; Teltow, Germany) with a sonotrode of 14 mm in diameter. The homogenization time was 4 min at an ultrasonic power of 54 W using an external ice bath as temperature control. After obtaining the emulsion, 180 mL of water was added to induce diffusion of solvent and aggregation of polymer with the formation of the nanoparticles. The diffusive process was carried out under the same conditions as the emulsion formation. Finally, the organic solvent was removed with a vacuum rotary evaporator (HB10; IKA^®^ Works, Inc.; Wilmington, NC, USA) at 30 °C and a reduced pressure of 66.6 Pa.

### 2.4. Nanocapsule Characterization

#### 2.4.1. Particle Size (Ps) and Polydispersity Index (PDI)

The dynamic light scattering technique was used for Ps and PDI measurement in a Malvern Zarasizer Nano ZS90 (Malvern Instruments Ltd.; Malvern, Worcestershire, UK) at a detection angle of 90° and a laser λ = 633 nm. One milliliter of each colloidal dispersion was diluted 10 times with distilled water. The measurements were performed in triplicate.

#### 2.4.2. Zeta Potential (ζ) of Nanoparticles

The electrophoretic movement of particles in dispersion was measured to obtain the zeta potential in a Zarasizer Nano ZS90 using polystyrene dispersions (ζ = −55 mV) as a reference. This parameter indicates the surface charge of the particles and the degree of repulsion between adjacent particles. All measurements were carried out in triplicate.

#### 2.4.3. Morphological Characterization of Nanocapsules

Polymeric NCs were placed on a glass slide in a refrigerated desiccator until the water evaporated entirely. The samples were coated with gold (≈2 nm) using a fine coat ion sputter deposition unit (JFC-1100 fine coat ion sputter; JEOL Ltd.; Akishima, Japan) and observed under a scanning electron microscope (SEM; JSM 5600 LV-SEM^®^ LV; JEOL Ltd.; Akishima, Japan) with a resolution of 5 nm. An electron beam of 28 kV and a chamber pressure of 12–20 Pa were the operational conditions.

#### 2.4.4. Determination of Nanocapsule Stability

The physical stability of the nanodispersions prepared with lemon essential oil and curcumin was analyzed using a Turbiscan^®^ Classic instrument (Toulouse, France). Five mL of each dispersion was placed in a cylindrical glass cell to ensure no air bubbles in the sample. The transmitted light was measured using a transmission detector, while the light scattered at 30° was detected using a backscattered sensor. The detection length was 55 mm, and measurements were obtained at 40 µm intervals along the sample. Measurements were carried out for four weeks. Measurements were taken at 0, 144, 193, 216, 482, 531, and 667 h (27.8 days). The Turbiscan Stability Index (TSI) was obtained from the backscattering data using Equation (1):

(1) $$\mathrm{TSI}=\sum_{i}\frac{\sum_{h}\left|{\mathrm{scan}}_{i}\left(h\right)-{\mathrm{scan}}_{i-1}\left(h\right)\right|}{H}$$

where ${\mathrm{scan}}_{i}\;(h)$ is the average backscattering for each measurement time (i), ${scan}_{i-1}\left(h\right)$ is the average backscattering for the previous time (i−1), and H is the sample height [30].

### 2.5. Application of Nanocoatings on Fresh-Cut Papaya

The selected papayas were washed and sanitized in a solution of iodine fruit detergent (2 g/L). Then, these were peeled, cut into approximately 1 cm cubed pieces, and immersed in a CaCl_2_ solution (10 g/L) for 3 min. Afterward, the papaya pieces were drained for 3 min and immersed in the different nanodispersions for 3 min. The evaluated treatments were: nanocapsules of lemon essential oil/PCL (NC L/PCL), nanocapsules of lemon essential oil/EC (NC L/EC), nanocapsules of curcumin/PCL (NC C/PCL), and nanocapsules of curcumin/EC (NC C/EC). In addition, fresh-cut papaya without any treatment was considered as the control treatment. The fresh-cut papaya was packaged in crystalline polypropylene cups (approximately 100 g per cup) and stored at 4 °C for 17 days.

### 2.6. Respiration Rate of Fresh-Cut Papaya Treated with Nanodispersions

The respiration rate was determined using the static method reported by Wang et al. (2009) [31] and Iqbal et al. (2008) [32] in the treated and stored papaya. The CO_2_ measurements were determined by measuring the headspace gas using a needle inserted through the container lid and analyzed using an O_2_/CO_2_ analyzer (Quantek Instruments model 905; Grafton, Massachusetts, USA) to obtain the volumetric fraction of CO_2_ and O_2_ inside the container. The CO_2_ production was calculated based on the difference in CO_2_ concentrations at different time intervals. The measurements were conducted during the storage period, in triplicate, and expressed as follows for CO_2_:

(2) $${RCO}_{2}=\frac{\left({yCO}_{2}-{y_{i}CO}_{2}\right)}{\left(t-t_{i}\right)}*\frac{V_{f}}{W}$$

where $yiCO_{2}$ is the initial concentration in the mixture (volumetric fraction), $yCO_{2}$ is the ${CO}_{2}$ concentration at any other time, t is any non-zero time expressed in hours $\left(t_{i}=0\right)$, $R{CO}_{2}$ is the ${CO}_{2}$ production rate, W is the mass of the product (kg), and $V_{f}$ is the volume (mL) inside the container.

The O_2_ consumption rate was calculated as Equation (3):

(3) $${RO}_{2}=\frac{\left({yO}_{2}-{y_{i}O}_{2}\right)}{\left(t-t_{i}\right)}*\frac{V_{f}}{W}$$

where $RO_{2}$ is the $O_{2}$ consumption rate, $yiO_{2}$ is the initial concentration in the mixture (volumetric fraction), $yO_{2}$ is the $O_{2}$ concentration at any other time, t is any non-zero time expressed in hours $\left(t_{i}=0\right)$, W is the mass of the product (kg), and $V_{f}$ is the volume (mL) inside the container.

### 2.7. Color Determination

The coloration of the treated and untreated papaya was determined by obtaining the values of L*, a*, and b* using a Minolta CM-600 colorimeter calibrated with L* = 57.79, a* = −1.09, b* = 7.57. Color measurements were taken on the cut and treated surface of the fresh-cut papaya (on any side of the cube) in triplicate during the storage period.

### 2.8. Determination of Firmness in Fresh-Cut Papaya

The firmness of the stored papaya was measured using a texture analyzer (CT3 Texture Analyzer; Brookfield AMETEK; Middleborough, MA, USA). The cut papaya was penetrated using a stainless steel flat-bottomed cylindrical probe with a diameter of 4 mm at a speed of 1 mm/s and a target depth of 5 mm. The measurements were performed in triplicate.

### 2.9. Measurement of Pectin Methylesterase (PME) Activity

The extraction of PME enzymes from papaya was performed following the protocol described by Zambrano-Zaragoza et al. (2014) [33]. Briefly, 20 g of fresh-cut papaya was homogenized with a NaCl solution (2 M). The homogenate was stirred for 10 min and then centrifuged at 7277× *g* for 20 min, with the supernatant containing the enzymatic extract. For the determination of PME activity, the protocol described by Hagerman and Austin (1986) [34] was followed. Briefly, in a spectrophotometric cell, 1 mL of citrus pectin (10 g/L), 580 mL of water, 200 µL of bromothymol blue (0.1 g/L), and NaCl (0.2 M) were added. Adding 200 µL of enzymatic extract initiated the reaction, and the decrease in absorbance at 640 nm (Genesys 10 UV-Vis; Thermo Fisher Scientific Inc.; Waltham, MA, USA) was measured for 3 min. The number of µmoles of released acid due to pectin methylesterase action was determined from a galacturonic acid standard curve following the treatment described by Hagerman and Austin (1986) [34].

### 2.10. Evolution of Polyphenol Oxidase (PPO) Activity

The polyphenol oxidase (PPO) was obtained by the methodology described by Galindo-Pérez et al. (2015) [32]. Briefly, 20 g of fresh-cut papaya was homogenized with 20 mL of a phosphate buffer solution (0.2 M; pH = 7.0). The homogenate was stirred for 10 min and then centrifuged at 7277× *g* for 20 min. The supernatant obtained was used as the enzymatic extract for measuring PPO activity. The PPO activity was determined by a reaction mixture of 2.8 mL citrate-phosphate buffer solution (0.2 M; pH = 6.5) containing catechol (50 mM) and 200 µL of enzymatic extract. The solution was gently stirred, and the increase in absorbance at 420 nm (Genesys 10 UV-Vis; Thermo Fisher Scientific Inc.; Waltham, MA, USA) was measured. One unit of polyphenol oxidase activity was defined as the change in absorbance per minute (0.001 Abs/min). The measurements were performed in triplicate.

### 2.11. Protein Determination

The analytical determination of protein in the enzymatic extracts was performed using the technique proposed by Bradford (1976) [35]. For this purpose, 100 µL of the enzymatic extract was mixed with 5 mL of Bradford reagent. The mixture was gently agitated and kept in the dark for 10 min, after which the absorbance was measured at 595 nm (Genesys 10 UV-Vis; Thermo Fisher Scientific Inc.; Waltham, MA, USA) using a bovine serum albumin standard curve under the same conditions.

### 2.12. Total Phenolic Compound Measurement

The extraction of total phenols was performed according to the technique reported by Waterhouse (2002) [36]. First, 20 g of fresh-cut papaya was homogenized with 20 mL of a methanol–water mixture (95%). The resulting solution was centrifuged at 7277× *g* for 20 min, and the supernatant contained the phenolic mixture. The quantification of total phenols was performed using the Folin–Ciocâlteu method. Briefly, 20 µL of the phenolic extract was mixed with 1.8 mL of water and 100 µL of Folin reagent and agitated for 5 min; then 300 µL of sodium carbonate was added. The mixture was kept in the dark for 1 h. The content of phenols was determined spectrophotometrically at 765 nm (Genesys 10 UV-Vis, Thermo Fisher Scientific Inc., USA) using a previously prepared gallic acid standard curve (y = 0.0009x + 0.0153; R^2^ = 0.996). The results were expressed as gallic acid equivalents (GAE) per 100 g of fruit. The determinations were performed in triplicate.

### 2.13. Statistical Analysis

An analysis of variance (ANOVA) was conducted to analyze the effect of the different treatments on papaya preservation. The significance of the differences was determined using Tukey’s multiple comparisons tests. Additionally, a Dunnett’s mean comparison test was used to assess the effect of each treatment compared to the control group [37]. Differences were considered significant with *p*-values < 0.05.

## 3. Results

### 3.1. Characterization of Nanoparticles

#### 3.1.1. Particle Size, Polydispersity Index, and Zeta Potential

Table 1 presents the results of NC particle size, polydispersity index, and zeta potential. The nanoparticles had particle sizes in the nanoscale range, with the NC L/PCL system having the smallest size. The PDI, with values from 0.126 to 0.246, indicates monodispersed systems with a narrow size distribution. The NC L/PCL and NC L/EC had PDI ≤ 0.150. The zeta potential of NCs formed ranged from −5.80 to −12.43 mV.

#### 3.1.2. Morphology of Nanocapsules

**Figure 1:**
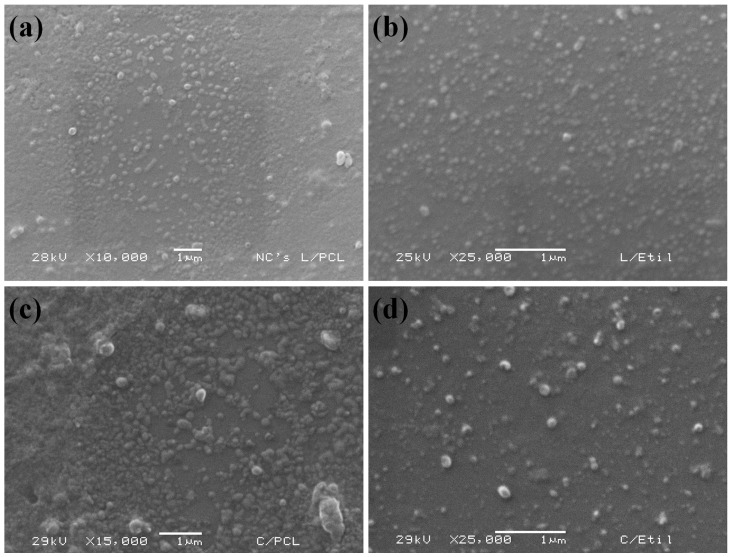
SEM micrographs of the different dispersions formed using the emulsification–diffusion method coupled with ultrasound: (**a**) NC L/PCL; (**b**) NC L/EC; (**c**) NC C/PCL; (**d**) NC C/EC.

Figure 1 shows the micrographs of the nanocapsules, showing that all systems had sizes ≤ 500 nm and spherical shapes. NC L/PCL and NC L/EC sizes are consistent with dynamic light scattering results (200 nm). Furthermore, NC C/PCL and NC C/EC exhibited spherical structures with Ps ranging from 150 to 200 nm.

#### 3.1.3. Instability Mechanism of Nanocapsules

Table 2 presents the instability mechanism of the NCs and the migration velocities of nanoscale particles in suspension obtained by the Turbiscan^®^ instrument. The NC L/PCL and NC C/PCL, as well as NC C/PCL and NC C/EC, were stable at room temperature during 667 h of storage. No significant changes in backscattering were observed; sedimentation was identified as the instability mechanism in all cases. This behavior is related to the calculated migration velocities of the particles. The NC L/EC and NC L/PCL had migration velocities in the bottom of 0.023 and 0.010 µm/min, respectively. The migration velocities in the top container for lemon oil NCs were 0.015 and 0.013 µm/min, demonstrating the high physical stability of the nanocapsules.

The NCs prepared with a curcumin oil core also showed good stability, as no significant changes in backscattering were observed during 4 weeks of storage at room temperature. The migration velocities in the bottom for NC C/EC and NC C/PCL were 0.011 and 0.009 µm/min, respectively.

#### 3.1.4. TSI of the Nanocapsules

**Figure 2:**
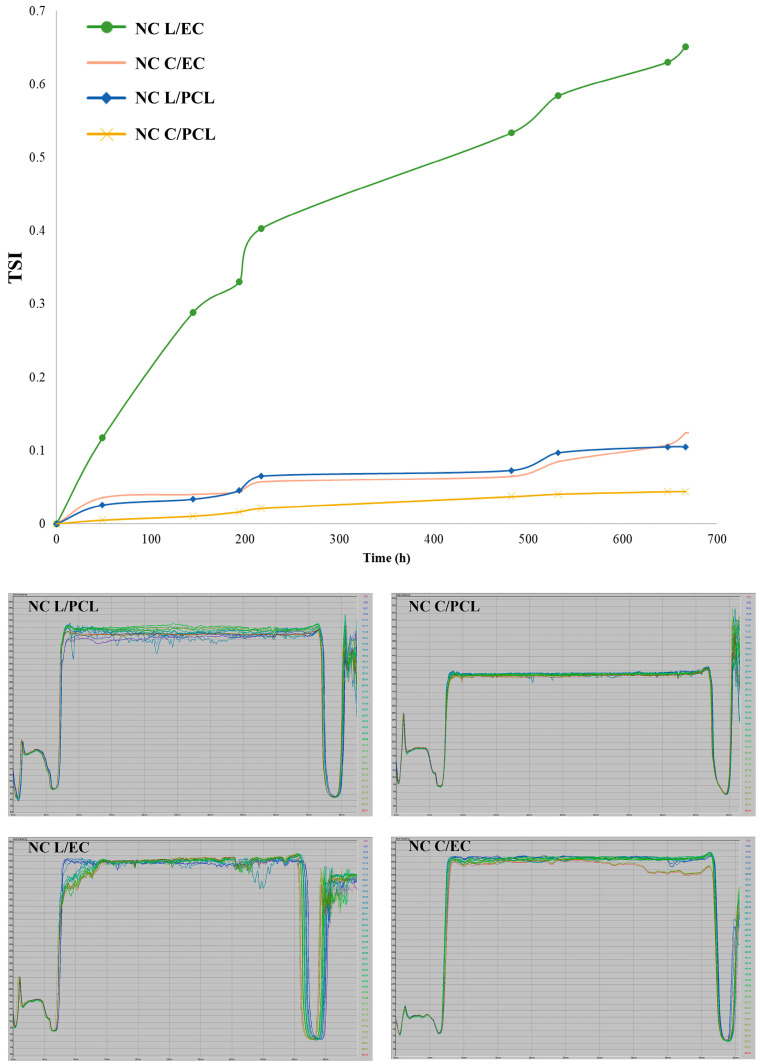
TSI of lemon oil and curcumin nanocapsules prepared with EC or PCL as barrier polymers.

Figure 2 shows the TSI values determined from the backscattering data measured by the Turbiscan^®^ equipment for lemon essential oil and curcumin NCs. All nanoscale systems presented TSI values lower than 1.0 after 27 days of storage at room temperature, suggesting high stability because TSI values close to zero indicate excellent stability. The TSI values for NC C/EC and NC C/PCL at the end of the measurement period were 0.10 and 0.044, respectively. Meanwhile, the NC L/EC had a TSI of 0.65 and NC L/PCL reached TSI values of 0.12.

### 3.2. Rate of CO_2_ Production in Papayas Treated with Nanocapsules

**Figure 3:**
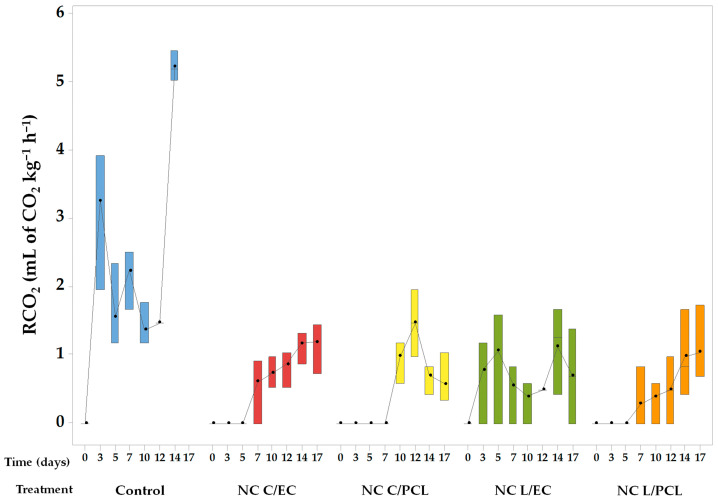
CO_2_ production rate of papaya treated with nanocapsules containing lemon oil or curcumin and PCL or EC as biopolymers.

Figure 3 presents the values for the rate of CO_2_ production of papaya as a function of the treatments used. The highest rate of CO_2_ production was observed in the control papaya batch, which had a significant increase during the first three days, with an average value of 3.27 mL of CO_2_ kg^−1^ h^−1^. This rate was maintained practically during the following days, and at the end of the storage period (day 14), another increase in the respiration rate was observed, reaching an average value of 5.23 mL of CO_2_ kg^−1^ h^−1^.

In contrast, in the papaya treated with NCs, a decrease in the rates of CO_2_ formation was observed in all cases. The statistical analysis demonstrated significant differences (*p* < 0.05) compared to the control batches of papaya. For papaya coated with NC L/EC, it was observed that the rate of CO_2_ production was slightly higher than 1.0 mL of CO_2_ kg^−1^ h^−1^ on days 7 and 14. The papaya treated with NC L/PCL exhibited a CO_2_ production rate of zero during the first five days of storage. However, the rate gradually increased after that, showing an upward trend in CO_2_ production. It reached a maximum value of 1.04 mL of CO_2_ kg^−1^ h^−1^ on day 17 of storage.

Similarly, papaya coated with NC C/EC and NC C/PCL showed no changes in CO_2_ production during the initial days until 7 and 10 days, respectively. Afterward, the CO_2_ production rate increased, reaching a CO_2_ production of 1.19 mL of CO_2_ kg^−1^ h^−1^ for papaya treated with NC C/EC and 1.45 mL of CO_2_ kg^−1^ h^−1^ for papaya treated with NC C/PCL.

### 3.3. Effect of Nanoparticles on the Rate of O_2_ Consumption

**Figure 4:**
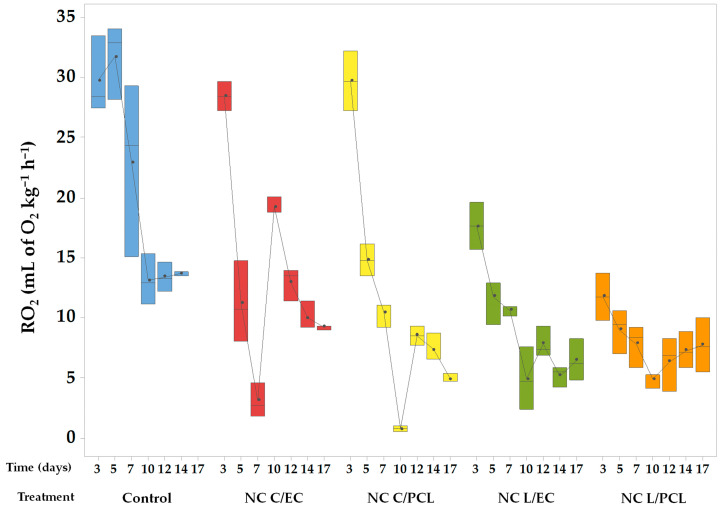
Oxygen consumption rate of papaya treated with nanoparticles containing lemon oil or curcumin.

Figure 4 presents the behaviors of the rate of O_2_ decrease in the headspace of containers with papaya under different treatments. It is worth noting that all systems had a significant effect (*p* < 0.05) on the rate of O_2_ consumption.

The control system exhibited high O_2_ consumption values during the first five days, with average rates of around 30 mL of O_2_ kg^−1^ h^−1^. The trend then decreased, and the rate of O_2_ consumption dropped to 13.72 mL of O_2_ kg^−1^ h^−1^ on the 14th day of testing. The nanocoatings of NC L/EC and NC L/PCL showed the lowest rates of O_2_ consumption, with initial values of 17.63 and 11.75 mL of O_2_ kg^−1^ h^−1^, respectively. Moreover, the rate of O_2_ consumption decreased during the remaining storage period, with O_2_ consumption rates of 6.45 mL of O_2_ kg^−1^ h^−1^ for NC L/EC and 7.72 mL of O_2_ kg^−1^ h^−1^ for NC L/PCL. The results were statistically significant compared to the control system. Meanwhile, the O_2_ consumption rates were 28.47 mL of O_2_ kg^−1^ h^−1^ for the NC C/EC treatment and 29.71 mL of O_2_ kg^−1^ h^−1^ for the NC C/PCL treatment during the first three days, gradually decreasing over the storage period to reach values of 9.22 and 4.91 mL of O_2_ kg^−1^ h^−1^, respectively.

### 3.4. pH and Acidity of Papaya Treated with Nanosystems

Table 3 presents the results obtained for the pH and acidity determinations of papaya treated with lemon oil and curcumin NCs using EC and PCL as barrier polymers during 17 days in cold storage.

The control samples showed a decrease in pH throughout the storage period, with an average pH value at the end of 4.97. Additionally, the acidity showed slight variations compared to its initial condition, resulting in final values of 0.416 mg citric acid/100 g of sample at the end of storage.

In papayas treated with NC L/EC, the pH barely changed, by 0.3, during storage. Similar behavior was remarked for papaya coated with NC C/EC, where the lowest pH value was found on day 7, with an average pH of 5.41. An increasing trend was observed in acidity during the first 12 days of storage, followed by a decrease. The final average value for the treatment with NC L/EC was 0.48 mg of citric acid/100 g of fruit, and for papaya coated with NC C/EC, it was 0.45 mg of citric acid/100 g. Statistical analysis showed significant differences (*p* < 0.05) between the encapsulated compounds.

The NC L/PCL and NC C/PCL showed a final value of 0.65 and 0.58 mg citric acid/100 g, respectively. In addition, the pH in papaya treated with NC L/PCL decreased drastically during the last week of storage, reaching a value of 4.8. The statistical analysis showed that pH and acidity exhibited significant differences between the untreated fresh-cut papaya, suggesting a protective effect against the deterioration of components in fresh-cut papaya.

### 3.5. Color Changes of Fresh-Cut Papaya Treated with Nanocapsules of Lemon Oil or Curcumin

**Figure 5:**
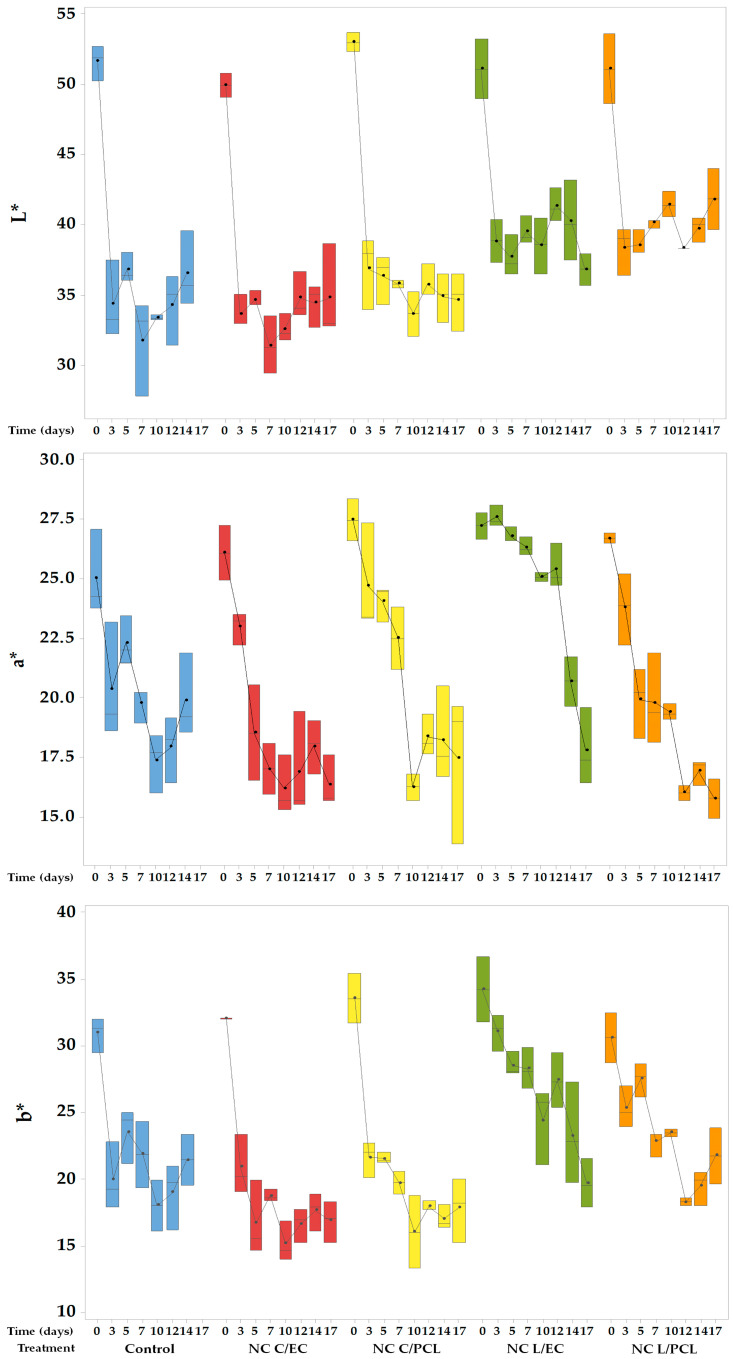
CIELab values (L*, a*, and b*) of the surface of fresh-cut papaya treated with different nanocapsules and storage under refrigeration.

Figure 5 shows the colorimetric values in CIELab coordinates of the surface of fresh-cut papaya treated with different nanodispersions and stored under refrigeration for 17 days. The L* value indicates the luminosity of the sample, where values close to 0 indicate black colors, while values close to 100 demonstrate white colors. This parameter in fruit preservation is related to the formation or degradation of compounds; for example, carotenoid degradation decreases the samples’ luminosity [38].

In control papaya, the initial average luminosity value was 51.59, decreasing drastically during the first 3 days and reaching an L* value of 34.34, which remained constant in the following days of storage, with an average L* value of 36.5 on day 14, corresponding to a 30% loss of luminosity compared to its initial state. The fresh-cut papaya treated with NC C/EC and NC C/PCL showed more significant changes in the L* values, decreasing rapidly during the first days of storage and remaining constant. The final loss of luminosity in fresh-cut papayas treated with NC C/EC and NC C/PCL was 30.06% and 30.24%, respectively, compared to their initial state, with no significant differences (*p* > 0.05) between the NCs containing curcumin and the control system. In contrast, the NC L/EC and NC L/PCL treatments showed luminosity losses of 18.2% and 27.9%, respectively, compared to their initial state. Furthermore, ANOVA statistical analysis revealed significant differences (*p* < 0.05) among the encapsulated oily materials. Additionally, Dunnett’s test indicated significant differences (*p* < 0.05) between the lemon oil nanoparticles and the control.

Figure 5 shows the a* value obtained from the surface of the papaya. The a* value indicates the change from red (positive) to green (negative) [38]. In the untreated control papaya, the a* value showed a decreasing trend during the storage period, with a decrease rate of −0.68 a*/day, reaching an a* value of 19.89 at the end of the storage period, representing a 30.42% loss of red coloration. The fresh-cut papaya with NC C/EC showed a change rate of −0.82 a*/day from day 0 to day 12, while for the papaya with NC C/PCL, the change rate was −0.95 a*/day in the same time interval. Dunnett’s test did not show statistical differences compared to the control. In contrast, the treatment that maintained the a* value for a more extended period was composed of NC L/EC, where a smaller decrease in the a* value was observed, with a change rate of −0.33 a*/day, indicating that the treatment was able to preserve the red coloration in fresh-cut papaya. ANOVA showed statistically significant differences between NC L/EC and all other treatments.

Figure 5 shows box plots for the evolution of the b* values of fresh-cut papayas treated with nanodispersions in cold storage. The b* scale ranges from positive values, indicating yellow colors, to negative values, where colors tend to be blue [38]. In the papaya without any treatment (control), a rapid decrease in the b* value was observed during the first days of storage, with a rate of change of −1.66 b*/day. Then, the b* value remained relatively constant until day 14 of storage, with a 42.1% loss compared to its initial condition. In the papaya with NC C/EC, a rapid decrease in the b* value was observed during the first 12 days of storage, with a loss rate of −1.1 b*/day. After day 12, the rate of b* loss changed and reached a value of −0.25 b*/day.

Similarly, this behavior was noted in papaya treated with NC C/PCL, where the rate of change in the b* value during the first 12 days was −1.2 b*/day. The calculated percentage loss of the b* value on day 12 was 48.1% for NC C/EC and 46.5% for NC C/PCL. The treatments containing curcumin did not show significant differences but differed from the other tested systems.

Conversely, for the fresh-cut papaya coated with NC L/EC, a downward trend of the b* value was observed throughout the storage period, with a decrease rate of −0.72 b*/day. A final value of 19.66 was found, representing a loss of 8.78% in the b* value on day 12 and 34.5% on day 17. Also, in the fresh-cut papaya treated with NC L/PCL, a significant decrease in the b* value was seen during the first 12 days, with a change rate of −0.84 b*/day. Subsequently, the rate decreased to −0.11 b*/day with a loss of b* value of 29% compared to day 0. Both treatments showed significant differences compared to the control.

### 3.6. Changes in the Firmness of Fresh-Cut Papaya Treated with Different Nanosystems

**Figure 6:**
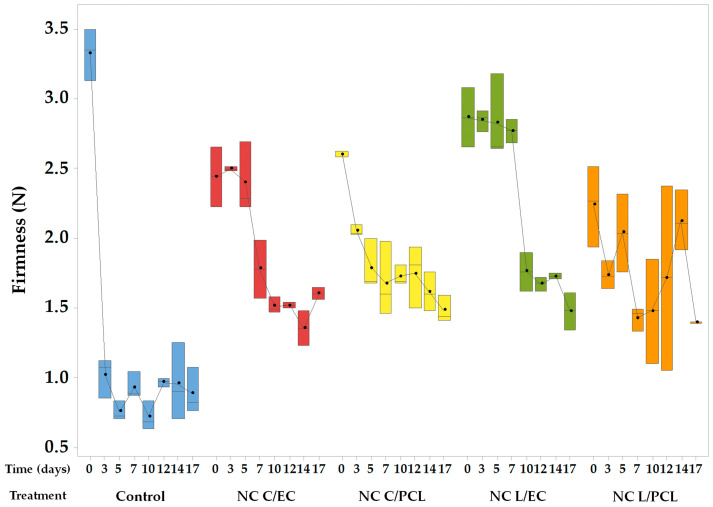
Firmness of papaya coated with different dispersions during 17 days of storage.

The firmness of the papaya was determined during 17 days of refrigerated storage; these results are presented in Figure 6. In the control papaya, firmness decreased rapidly during the first 3 days of storage. The average firmness on day zero was 3.35 N. In contrast, on day 3, it decreased to an average firmness of 1.07 N, indicating that the control papaya lost up to 68% of its initial firmness during the early storage period.

The firmness loss of fresh-cut papaya treated with NC L/EC compared to its initial value was only 9% on the 5th day. However, firmness decreased to 1.57 N on day 7, with a firmness loss percentage of 26% and reaching a final value of 36%. For the papaya treated with NC L/PCL, a firmness loss rate of 0.017 N/day was observed during the storage period, with a firmness of 1.54 N on day 17, representing a 32% decrease compared to its initial state. For the papaya coated with NC C/EC, a periodic decrease in firmness was observed, reaching a final value of 1.44 N, which represents a firmness loss of 39% compared to the initial state, reducing the percentage of loss by more than 20% compared to the untreated system. Meanwhile, in the papaya treated with NC C/PCL, firmness loss rates of 0.038 N/day were observed, resulting in a final firmness of 1.36 N with a 40% loss of firmness compared to day zero. Statistical analysis showed significant differences (*p* < 0.05) between the control system and the nanocapsule treatments containing lemon oil or curcumin, indicating firmness preservation for 17 days of refrigerated storage.

### 3.7. PME Activity of Fresh-Cut Papaya Treated with Different Polymeric Nanoparticles

**Figure 7:**
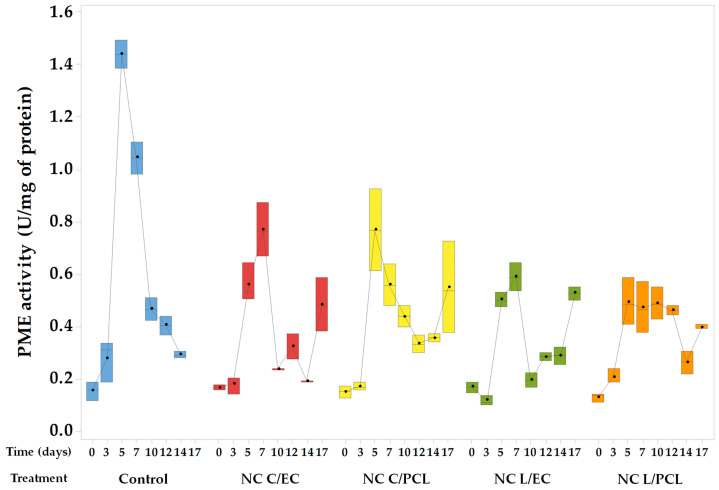
Changes in the activity of the PME enzyme in treated and stored papaya.

Figure 7 shows the values of papaya coated with NC L/EC, NC L/PCL, NC C/EC, and NC C/PCL, as well as the control samples of papaya without any treatment. The control batch of papaya exhibited an increasing trend in pectin methylesterase (PME) activity during the initial days of storage, correlating with a drastic loss of firmness. The maximum PME activity of 1.44 U/mg protein was reached on the fifth day of storage, which then decreased, reaching an activity of 0.29 U/mg protein on day 17.

In papaya coated with NC L/EC, an increase in PME activity was observed, reaching a maximum value on the fifth day of storage of 0.41 U/mg of protein. This level was maintained practically throughout the rest of the testing period. In the case of NC L/PCL, the PME activity was initially low during the early days of storage, with an average activity of 0.14 U/mg of protein. However, a considerable increase in activity was noted on the seventh day, with an activity of 0.59 U/mg of protein, followed by a decrease. Papayas treated with NC C/EC showed a maximum PME activity on day 5, with an average activity of 0.72 U/mg of protein. At the same time, NC C/PCL samples reached their maximum activity on day 7, with an activity of 0.76 U/mg of protein. Statistical analysis did not show significant differences among the evaluated nanometric treatments.

### 3.8. PPO Activity of Fresh-Cut Papaya Treated with Nanocapsule Systems

**Figure 8:**
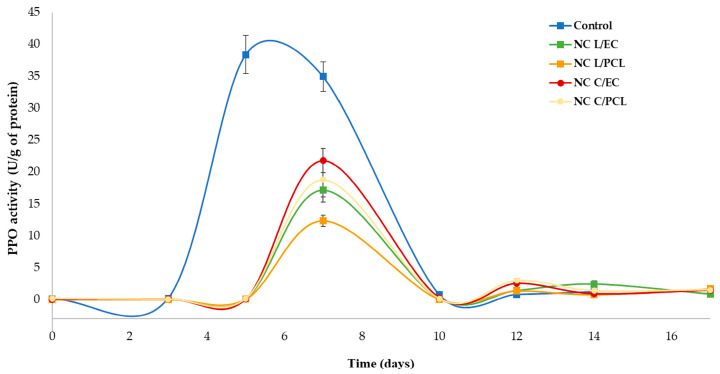
Evolution of PPO enzymatic activity in papaya coated with nanoparticles and stored under refrigeration.

Figure 8 presents the results obtained for PPO enzyme activity in fresh-cut papaya treated with nanoparticles. Dunnett’s multiple comparison tests revealed statistically significant differences between the nanoparticulate treatments and the control system, indicating that the treatments used decreased PPO activity. For the control system, it was found that PPO enzyme activity increased during days 5 and 7, reaching activity values of 35.53 and 32.68 U/g protein, respectively. Subsequently, PPO activity declined to values lower than 1 U/g protein. In the treatments of NC L/EC and NC L/PCL, an increase in PPO activity was observed on day 7, reaching values of activity of 17.14 and 12.35 U/g of protein, respectively, corresponding to inhibition percentages of 47.55% and 62.18%, showing statistically significant differences compared to the control system. In the case of fresh-cut papaya treated with NC C/EC and NC C/PCL, an increase in PPO activity was seen on day 7 of storage with an activity of 21.7 and 18.7 U/g of protein, with an inhibition percentage of PPO activity of 33.61% and 42.79%, respectively. The ANOVA showed significant differences compared to the control system (*p* < 0.05).

### 3.9. Changes in the Total Phenolic Content in Fresh-Cut Papaya Treated with Nanodispersions

**Figure 9:**
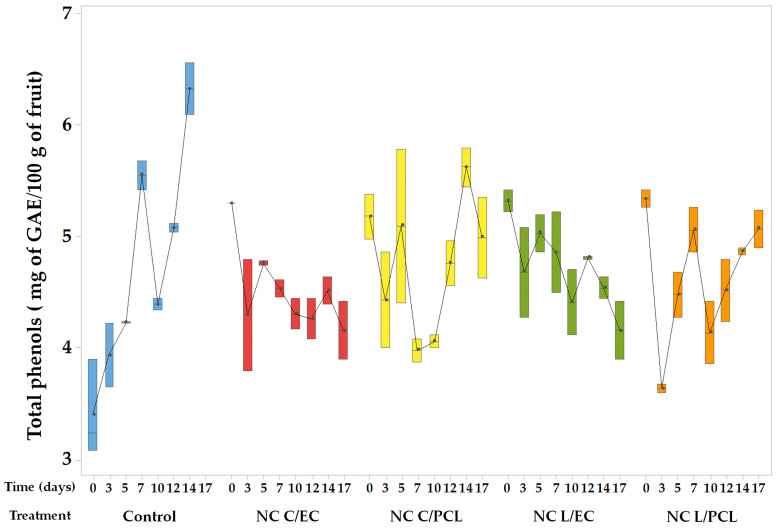
Total phenol content in fresh-cut papaya coated with nanodispersions and stored under refrigeration.

Figure 9 presents the results of total phenols in fresh-cut untreated papaya (control) and papaya coated with NCs of lemon oil and curcumin using EC or PCL as biopolymers. In the control system, an increase in the content of total phenols was observed, showing an upward trend throughout the storage period, with a production rate of phenolic compounds of 0.17 mg of gallic acid equivalents/day. The Dunnet test showed significant differences between the control papaya and the papaya coated with the nanodispersions.

The trend for papaya treated with NCs formed by EC containing lemon oil or curcumin showed a slight decreased concerning phenolic compounds. In papaya treated with NC L/EC, a decrease rate in the total phenols of 0.043 mg of gallic acid equivalents/day was observed. Meanwhile, NC C/EC had a 0.047 mg gallic acid equivalents/day rate. For nanoparticles containing either lemon oil or curcumin formed with PCL, less variation in the content of phenolic compounds was observed; however, statistical analysis did not show significant differences between the NCs prepared with PCL or EC.

## 4. Discussion

### 4.1. Ps, PDI, ζ, and Morphology of Nanocapsules

Table 1 shows that the PS of lemon oil and curcumin NCs using EC and PCL ranged from 87 to 116 nm. The NC L/EC and NC L/PCL had sizes of 116 nm and 87.57 nm, respectively. Hasani et al. (2018) [39] reported Ps of 339 and 553 nm for lemon essential oil nanocapsules with chitosan and OSA-starch, respectively. Moreover, a study of nanocapsules prepared with methyl methacrylate-styrene as barrier polymer showed particle sizes of 136 nm [40], coinciding with our report. The average Ps values for the NC C/EC and NC C/PCL were 115 nm and 100 nm, respectively, falling within the nanoscale range. A study on curcumin extract NCs of sodium caseinate reported a Ps of 165 nm [41]. Similarly, the nanoencapsulation of curcumin with different oils such as castor, soybean, and Miglyol oil exhibited particle sizes of 150 nm, 142.5 nm, and 206 nm, respectively [42].

The PDI of lemon oil and curcumin NCs was lower than 0.25 (Table 1), indicating a narrow distribution of particle sizes. In lemon essential oil NCs prepared by ionic gelation and freeze-drying, a PDI of 0.424 was reported when chitosan or modified starch was used [39]. Moreover, PDIs between 0.16 and 0.29 have been reported for curcumin NCs dissolved in different oils, implying that the prepared systems have good homogeneity [43]. Then, the results of this study showed their comportment.

The ζ of NCs (Table 1) is partially correlated with stability. However, due to the characteristics of non-ionic stabilizers, low zeta potentials are generally observed, where steric effects prevent the aggregation or flocculation of the nanoparticles, and the systems remained stable for 4 weeks under ambient temperature storage conditions. For lemon oil nanocapsules prepared with chitosan and starch, ζ-values of 10.58 mV have been reported when the chitosan/starch ratio was 0.5%/9.5% [39].

The micrographs of lemon oil and curcumin NCs obtained by SEM (Figure 1) show spherical and nanometric sizes from 150 to 200 nm. Similar results have been reported for nanocapsules containing fragrances for textile scenting using lemon oil as the main compound, with spherical nanocapsules ranging from 100 to 200 nm [40]. Nanoscale spherical systems with particle sizes between 150 and 200 nm were observed with SEM for curcumin/PCL NCs obtained using solvent displacement methodology [44]. Curcumin/EC NCs prepared by dialysis have been reported as spherical structures with a particle diameter of 282.9 nm [45].

Therefore, the emulsification–diffusion process using ultrasound homogenization is an excellent tool for preparing nanoscale systems capable of encapsulating essential oils and curcumin with a good size distribution and stability, which can be helpful for food preservation purposes.

### 4.2. Stability of Nanocapsules

Sedimentation was the main instability mechanism for lemon oil and curcumin NCs manufactured with EC and PCL. However, the low migration velocities indicated they were highly stable at room temperature during 27 days of storage. These results correlate with TSI because values less than 1.0 were obtained (Figure 2). This pattern is due to the steric effect of the stabilizer used to prevent flocculation or aggregation of nanoparticles [46]. According to different authors [47,48], particles in dispersion with zeta potentials close to zero are considered unstable. Stabilizers like PVA can stabilize nanodispersions due to steric effects that prevent the binding of one particle to another, avoiding aggregation and promoting the stability of the NCs in suspension. In a study of curcumin nanoparticles prepared with a zein–shellac mixture (1:1), TSI values ranged from 1.54 to 8.43, with higher stability [49]. For α-limonene-based emulsions and nanoemulsions, TSI values between 2.37 and 6.01 were found after 30 days of storage [50]. D-limonene emulsions showed TSI values ranging from 1 to 15, with emulsions more stable when using Pluronic PE9400 [51]. Therefore, the fabrication of NCs increases the stability of dispersions, helping to maintain the physicochemical stability of the encapsulated active agent. In addition, the emulsification–diffusion methodology with ultrasound homogenization effectively prepares NCs with good physical stability.

The migration velocity of NC L/EC at the base of the measurement cell was two times higher than that of the other prepared systems (Table 1), with TSI values 6 to 10 times higher (Figure 2). This behavior could be attributed to the interactions between EC and fractions with high water solubility of lemon oil. The lemon essential oil obtained through steam distillation is composed of poorly water-soluble compounds, such as limonene, γ-terpinene, and α- and β-pinene. It also includes compounds with higher water solubility, such as neral, geranial, neryl acetate, and geranyl acetate [52]. Compounds with higher water solubility can interact with the hydroxyl groups of EC through hydrogen bonding, which could potentially diminish the interactions between these compounds and the hydrophilic stabilizers (PVA and OSA-starch), resulting in minor stability of the NC L/EC. According to Zhao et al. (2018) [53], citrus-based emulsions (mandarin, sweet orange, and bergamot) were more stable as the content of polar compounds increased due to hydrophilic compounds being more prone to covering the hydrophilic stabilizer (Tween 80), thereby enhancing the emulsion’s interfacial properties and improving its stability.

### 4.3. Respiration Rate of Fresh-Cut Papaya Treated with Nanocapsules

The slicing of the tissues induces a cascade of metabolic changes in fresh-cut fruits, including an increase in respiration rate (O_2_ consumption and CO_2_ production). A significant reduction in respiration rate during the initial storage days contributes to the extended shelf-life of fresh-cut fruits. The reduction in respiration decreased ethylene production (a maturation hormone) and the downregulation of enzymes related to fruit degradation, such as pectin methylesterase, polygalacturonase, and polyphenol oxidase, among others. These mechanisms preserve firmness, color, and bioactive compounds like polyphenols [54]. Consequently, nanoparticle-based coatings can form a barrier that limits gas exchange, thus reducing the respiration rate in fresh-cut fruits [55].

The CO_2_ production and O_2_ consumption rates in the control papaya were significantly higher than in the papayas coated with the nanosystems (Figure 3 and Figure 4). This behavior is because of cuts that increase the surface area in the control papaya. Furthermore, barriers that limit gas permeability have been eliminated, contributing to an increase in the respiration rate of the papaya [56]. The papaya utilized the O_2_ inside the container to continue its metabolism, resulting in a high respiration rate and increased concentration of CO_2_ in the container’s headspace. Additionally, the onset of biodegradative metabolism leads to an increase in the concentration of CO_2_ caused by the need to produce energy (ATP) for survival [57,58].

In contrast, the nanocoating formed by NC L/EC, NC L/PCL, NC C/EC, and NC C/PCL showed a statistically significant reduction of CO_2_ production and O_2_ consumption rates compared to the control system, indicating that the nanoparticles form a coating on the surface of the fresh-cut papaya, limiting the diffusion of O_2_ and respiration of the product. This reduction in respiration helped to preserve the freshness, quality, and desirable attributes of the papaya, such as its texture, color, and flavor. Similar behavior was observed in chitosan nanoparticles encapsulating lemon essential oil applied as a coating on strawberries, where the O_2_ consumption rate was reduced compared to chitosan nanoparticles alone and the control system [59]. In whole cucumbers coated with chitosan nanoparticles encapsulating cinnamon essential oil, the respiration rate of the cucumbers decreased significantly compared to the uncoated cucumbers, with no statistically significant differences observed compared to the chitosan treatment without nanoparticles [60]. The ability of the NCs to slow down the respiration process can be attributed to their film-forming properties and ability to create a protective layer [61]. This layer acts as a barrier against oxygen and moisture, preventing oxidative reactions and the growth of microorganisms that contribute to the deterioration of the papaya.

### 4.4. pH and Acidity of Fresh-Cut Papaya Treated with Nanocapsules

The pH in the control papaya had a decrescent tendency, while after day 10, the acidity experienced a sharp reduction. The decrease in pH is attributed to the formation of organic acids due to the biotransformation of carbohydrates into organic acids and the growth of microorganisms on the papaya’s surface, which can produce organic acids. The reduction of acidity in control papaya is due to using organic acids such as citric acid and malic acid by enzymes during respiratory processes [62].

The NC L/EC and NC C/EC showed slightly varied pH and acidity content during storage. The slight differences in pH and acidity behavior are associated with nanosized treatments that can act as a barrier to gas exchange, reducing papaya respiration and thereby maintaining the carbohydrates that are not modified or used in respiratory processes. More minor changes in pH and fruit acidity have been observed for papaya coated with chitosan, implying that chitosan forms a barrier, reducing fruit respiration and increasing the stability of papaya components [62]. In contrast, for the NC L/PCL and NC C/PCL, an increase was observed throughout the storage period regarding acidity. While the NCs have a protective effect by reducing the respiration rate, PCL can degrade over time, forming carboxyl end groups that can decrease the acidity of the samples. The degradation of PCL occurs because the polymer in an aqueous environment can swell, causing water molecules to interact with the polymer, attacking the ester groups of PCL, leading to hydrolysis and the formation of terminal carboxyl groups. Furthermore, the hydrolysis of ester groups can be catalyzed by lipase enzymes found in papaya tissue [63].

### 4.5. Changes in the Color of Fresh-Cut Papaya Treated with Nanosystems

The control system showed a rapid decrease in the luminosity of the papaya surface (Figure 5). This behavior is attributed to the loss of physical barriers, allowing greater oxygen diffusion into the exposed tissue, leading to the decomposition of papaya components, especially carotenoids that give the fruit its red color. In addition, oxidation reactions of phenols increase, resulting in the darkening of the samples, as observed by González-Aguilar et al. (2009) [8] in fresh-cut ‘Maradol’ papaya. The systems containing lemon oil NCs maintained the papaya’s luminosity, as they could limit gas transfer, especially oxygen. As a result, the oxidation of carotenoids that give the fruit its red color and the formation of dark compounds caused by polyphenol oxidase enzyme were reduced. Moreover, incorporating liposoluble compounds in edible coatings can modify the oxygen permeability properties of the formed coatings [64]. Furthermore, for fresh-cut ‘Formosa’ papaya coated with montmorillonite (TP = 100 nm) supported by a coating formed by a corvina protein isolate, it was observed that the use of nanoscale dispersions significantly reduced the loss of luminosity of the papaya [21].

The decrease in a* value in the control papaya (Figure 5) is attributed to the oxidation of carotenoids that give pigmentation to the fruit due to the absence of barriers that can interfere with oxygen distribution within the papaya tissues [65]. Papayas treated with NC C/EC and NC C/PCL did not differ significantly from the control. This behavior is associated with the degradation of curcumin, which leads to a loss of coloration [66]. In comparison, the NC L/EC treatment exhibited the slightest fluctuations in the a* value, suggesting that the treatment generated a barrier between the cut surface of the papaya and the environment, thereby reducing the entry of oxygen into the tissues and minimizing color changes in the product.

The rapid diminishing of the b* value in the control papaya (Figure 5) is due to the exposure of the papaya tissues and their components to oxygen, which can lead to oxidation or degradation phenomena. In papaya packaged in PVC bags with 15 microperforations, a percentage decrease in the b* value of 36.94% was noted after 9 days of storage compared to its initial state. Moreover, an increase in browning for the system with a higher number of microperforations was observed, leading to greater oxygen exposure, subsequent activation of polyphenol oxidase, and the formation of melanin [67]. In the papaya with NC C/EC and NC C/PCL, the percentage of b* value loss was higher compared to the control system; this additional decrease in b* value may be due to the decomposition of curcumin constituents that give it its characteristic yellow color, such as demethoxycurcumin, curcumin, and bisdemethoxycurcumin, which can be easily degraded by light [68], taking several days [69] and resulting in the loss of yellow coloration in the samples. Minor changes in the b* value were obtained with the NCs containing lemon oil, suggesting that nanoparticles can limit the oxygen exchange between the cut papaya and the environment, allowing the product’s sensory characteristics to be maintained. A lower presence of oxygen in the cut tissues reduces the oxidation of color-giving compounds such as carotenoids. It decreases the activities of enzymes involved in forming brown compounds, such as polyphenol oxidases [58].

### 4.6. Firmness of Fresh-Cut Papayas Treated with Nanocapsules

Firmness is the maximum penetration force of a texturometer probe capable of breaking the surface of fresh-cut papaya. Firmness is determined by the physical anatomy of the tissue, such as size, shape, cellular arrangement, cell wall thickness, and cell cohesion degree [58].

The significant loss of firmness in the control papaya (Figure 6) is due to the higher activity of pectolytic enzymes in the control papaya that modified and hydrolyzed the pectins of the cell wall. This behavior has been widely studied in papaya. For example, in ‘Sunrise Solo’ papaya without any treatment, a firmness loss of approximately 50% was observed in the first two days of storage, decreasing from 4.7 to 2.5 N [70]. Likewise, González-Aguilar et al. (2009) [8] observed a 64% loss of firmness in fresh-cut ‘Maradol’ papaya without any treatment during the first 3 days of refrigerated storage. These are similar results to those reported in this study.

For NC treatments, up to 40% firmness loss was achieved. The coating formed on the papaya surface decreases the metabolism activity of pectic enzymes related to changes in papaya firmness. For fresh-cut ‘Sinta’ papaya coated with nanochitosan, it was observed that the nanosystems could reduce papaya firmness loss [71]. For fresh-cut papaya coated with nanocomposites composed of whitemouth croaker (*Micropogonias furnieri*) protein isolates and montmorillonite, a firmness loss of only 17% was found compared to untreated samples that had a firmness loss of 69.76% after 12 days of refrigerated storage [21]. Furthermore, the antioxidant capacity of lemon oil and curcumin could help maintain the structure of the cell membrane. While the rapid appearance of oxidizing species affects the structure of the cell membrane, the antioxidants contained in the NCs could limit the oxidation of phospholipids and thus maintain the physical structure of fresh-cut papaya, as has been observed by Velderrain-Rodríguez et al. (2015) [72] in papaya treated with antioxidants obtained from mango.

### 4.7. PME Activity in Fresh-Cut Papaya Treated with Nanoparticles

As shown in Figure 7, the control samples exhibited the highest PME activities; this behavior is attributed to the induction of ripening processes in the control papaya caused by cuts, which, in turn, increase the activities of pectolytic enzymes such as PME and polygalacturonase, leading to the modification and hydrolysis of pectins [58]. González-Aguilar et al. (2009) [8] observed that fresh-cut ‘Maradol’ papaya without any treatment exhibited a rapid increase in PME activity during the first 3 days of storage compared to papaya treated with chitosan. In addition, tissue damage results in the rapid release of linolenic and linoleic acids from phospholipids in cell membranes due to lipoxygenases, which act as chemical signalers in synthesizing cell wall-related enzymes. Moreover, the hydrolysis of pectins caused by the activity of pectolytic enzymes like PME and polygalacturonase results in the release of oligogalacturonides, leading to the overexpression of the PME enzyme [73].

In contrast, in the papayas treated with NCs, PME activity was partially inhibited. This behavior indicates that the NCs incorporated on the cut surface of the papaya can generate a coating that reduces the respiration rate of the papaya and the ethylene production and inhibits the signaling cascades involved in the synthesis and activation of pectinolytic enzymes such as PME [74].

### 4.8. PPO Activity of Fresh-Cut Papaya Treated with Nanoparticles

As shown in Figure 8, the activity of the PPO enzyme was much higher in control papayas than in the papayas treated with nanodispersions. This behavior is associated with the absence of any physical barrier that limits the transport of O_2_ inside the papaya, allowing more significant interaction of O_2_ with PPO and phenolic compounds found in the fruit. In untreated papaya, Arjun et al. (2015) [63] found that the PPO activity increased during the initial days and decreased after reaching peak activity. The PPO activity was significantly higher than that found in papaya treated with a chitosan–soy coating, indicating that the lower PPO activity is due to the protective effect of the coating, which limits the oxygen supply to the tissue, thereby reducing PPO activity.

In addition, the treatments with nanocapsules showed up to 63% PPO inhibition. These results are attributed to the fact that the nanoparticles can form a coating on the cut surface of the papaya. This coating dramatically limits the transfer of gases between the papaya and the environment. As a result, a modified atmosphere is generated within the fruit tissue, leading to decreased PPO activity, fewer color changes due to a decrease in the formation of dark compounds, and the preservation of biologically valuable components in fresh-cut papaya. A decrease in PPO activity has also been found for apples treated with nanochitosan, which is related to the oxygen barrier properties of chitosan [75]. The NC C/EC and NC C/PCL treatments were less effective in limiting gas transfer than the NC L/EC and NC L/PCL systems. A higher amount of oxygen in fresh-cut papaya promotes the activation of PPO enzymes, increasing browning development and decreasing the bioactive compounds. Furthermore, essential oils can inhibit PPO activity, as Eissa et al. [65] demonstrated in apple juice, where lemon grass oil extract showed inhibition of 92%. Regarding using nanostructures to decrease PPO activity, it has been found that in fresh-cut ‘Red Delicious’ apples treated with α-tocopherol nanocapsules, PPO enzyme activity was delayed compared to the CaCl_2_ treatment [76].

### 4.9. Total Phenolic Content in Fresh-Cut Papaya Treated with Nanocapsules

The increasing trend in the total phenolic content in the control papaya (Figure 9) is attributed to the degradation of both the cell wall and the cell membrane, generating species such as oligogalacturonides from the hydrolysis of pectins or free fatty acids because of the hydrolysis of papaya cell membrane phospholipids, which serve as signals for the production and activation of new enzymes involved in protection mechanisms against pathogen attacks. Oligogalacturonides induce the formation and activation of enzymes such as phenylalanine ammonia-lyase, which is the crucial enzyme in the phenylpropanoid (or shikimic acid) pathway, producing a wide variety of phenolic compounds and lignin that obstruct pathogen attack. Likewise, the chalcone enzyme metabolizes phytoalexin production, which has antimicrobial activity [54].

Alternatively, the content of phenolic compounds in fresh-cut papaya treated with NCs had slight variations over 17 days of refrigerated storage. This behavior is attributed to the excellent interaction between the pectin in the papaya cell wall and the EC of NCs [77] that were deposited on the surface of the fruit, reducing the degradation of pectins and signaling reactions involved in the synthesis of phenolic compounds. Also, PCL NCs can integrate into the fruit surface, forming a coating that minimizes the degradation of fresh-cut papaya. Furthermore, the presence of antioxidant compounds such as lemon oil and curcumin promotes the recycling of the antioxidant activity of phenolic compounds [78]. Similar behavior has been observed in recent studies where α-tocopherol/PCL nanocapsules reduce the initial respiration rate and the enzyme activities of PME and phenylalanine ammonia-lyase in fresh-cut ‘Red Delicious’ apples [76,79].

## 5. Conclusions

The main advantage of nanoencapsulation is that it allows the incorporation of many active substances that, due to their chemical or physicochemical properties, are difficult to mix with food matrices. Also, the encapsulated active compounds are protected from degradation reactions, and controlled release of the encapsulated compounds can also be achieved. Therefore, nanocapsules manufactured with approved compounds for food use are an interesting option for preserving fresh-cut fruits.

NCs containing curcumin and lemon essential oil were obtained by the emulsification–diffusion method coupled with ultrasound and using EC or PCL as barrier biopolymers. The systems exhibited particle sizes below 150 nm, polydispersity indices below 0.2, and zeta potentials higher than −10 mV. The instability mechanism observed in the lemon oil and curcumin NCs was sedimentation. However, they remained stable for 27 days when stored at room temperature. The EC- and PCL-based NCs of lemon oil and curcumin showed a significant reduction in the respiration rate of fresh-cut papaya during 17 days of storage. The EC-based NCs displayed less variation in the acidity and pH of fresh-cut papaya. The NCs effectively mitigated physical changes associated with the degradation of fresh-cut papaya, with particular attention given to the treatment with lemon oil/EC nanocapsules, demonstrating better color and firmness retention. Furthermore, all nanosystems decreased PPO and PME enzymatic activities, which correlated with the retention of quality characteristics and total phenolic content in the fresh-cut papaya. The lemon oil nanocapsules and the curcumin-based nanocapsules employing EC and PCL as biopolymers may be extended to conserve various fresh-cut fruits and vegetables.

## Figures and Tables

**Table 1:** Nanocapsule characterization of lemon essential oil and curcumin extract using EC and PCL as encapsulant polymers.

System	Ps (nm)	PDI	ζ (mV)
NC L/EC	116 ± 0.72	0.126 ± 0.02	−7.52 ± 0.53
NC L/PCL	87.57 ± 0.34	0.142 ± 0.01	−12.43 ± 0.98
NC C/EC	115.73 ± 0.83	0.246 ± 0.01	−6.15 ± 1.04
NC C/PCL	100.18 ± 0.98	0.167 ± 0.01	−5.80 ± 0.78

Ps = particle size; PDI = polydispersity index; ζ = zeta potential.

**Table 2:** Instability mechanism and migration velocity of lemon oil and curcumin nanocapsules manufactured with PCL or EC as barrier polymers.

System	Instability Mechanism	Average Migration Rate in the Bottom (µm/min)	Average Migration Rate in the Top (µm/min)
NC L/EC	Sedimentation	0.023	0.015
NC L/PCL	Sedimentation	0.010	0.013
NC C/EC	Sedimentation	0.011	0.012
NC C/PCL	Sedimentation	0.009	0.010

**Table 3:** Changes in pH and acidity of papaya treated with lemon oil and curcumin nanocapsules using EC and PCL as barrier polymers.

Time		Control	NC L/EC	NC L/PCL	NC C/EC	NC C/PCL
Day 0	pH	5.20 ± 0.01	5.25 ± 0.03	5.23 ± 0.01	5.11 ± 0.04	5.20 ± 0.04
	Acidity (mg citric acid/100 g)	0.518 ± 0.05	0.532 ± 0.01	0.525 ± 0.03	0.534 ± 0.07	0.538 ± 0.06
Day 3	pH	5.21 ± 0.08	5.62 ± 0.02	5.45 ± 0.02	5.67 ± 0.02	5.33 ± 0.06
	Acidity (mg citric acid/100 g)	0.395 ± 0.02	0.529 ± 0.02	0.431 ± 0.02	0.375 ± 0.02	0.533 ± 0.02
Day 5	pH	5.30 ± 0.02	5.55 ± 0.09	5.54 ± 0.05	5.53 ± 0.04	5.59 ± 0.04
	Acidity (mg citric acid/100 g)	0.363 ± 0.05	0.497 ± 0.02	0.395 ± 0.02	0.400 ± 0.02	0.463 ± 0.02
Day 7	pH	5.23 ± 0.02	5.37 ± 0.05	5.37 ± 0.04	5.41 ± 0.05	5.38 ± 0.03
	Acidity (mg citric acid/100 g)	0.405 ± 0.02	0.568 ± 0.01	0.494 ± 0.02	0.535 ± 0.01	0.509 ± 0.03
Day 10	pH	5.35 ± 0.01	5.25 ± 0.01	5.52 ± 0.01	5.40 ± 0.07	5.48 ± 0.01
	Acidity (mg citric acid/100 g)	0.469 ± 0.04	0.520 ± 0.01	0.689 ± 0.02	0.608 ± 0.03	0.546 ± 0.03
Day 12	pH	5.16 ± 0.04	5.16 ± 0.06	5.35 ± 0.12	5.48 ± 0.03	5.46 ± 0.02
	Acidity (mg citric acid/100 g)	0.416 ± 0.03	0.560 ± 0.02	0.515 ± 0.03	0.511 ± 0.03	0.469 ± 0.02
Day 14	pH	5.08 ± 0.03	5.27 ± 0.04	4.94 ± 0.05	5.40 ± 0.02	5.30 ± 0.04
	Acidity (mg citric acid/100 g)	0.427 ± 0.05	0.417 ± 0.01	0.592 ± 0.02	0.428 ± 0.02	0.546 ± 0.03
Day 17	pH	4.97 ± 0.05	5.56 ± 0.04	4.86 ± 0.04	5.47 ± 0.04	5.29 ± 0.04
	Acidity (mg citric acid/100 g)	0.416 ± 0.03	0.480 ± 0.03	0.656 ± 0.02	0.449 ± 0.01	0.576 ± 0.03

## Data Availability

The data presented in this paper are available upon request from the corresponding author.

## References

[1] Wilson M.D., Stanley R.A., Eyles A., Ross T. (2019). Innovative Processes and Technologies for Modified Atmosphere Packaging of Fresh and Fresh-Cut Fruits and Vegetables. Crit. Rev. Food Sci. Nutr..

[2] Hu W., Sarengaowa W., Guan Y., Feng K. (2022). Biosynthesis of Phenolic Compounds and Antioxidant Activity in Fresh-Cut Fruits and Vegetables. Front. Microbiol..

[3] Botondi R., Barone M., Grasso C. (2021). A Review into the Effectiveness of Ozone Technology for Improving the Safety and Preserving the Quality of Fresh-Cut Fruits and Vegetables. Foods.

[4] Dotto J.M., Abihudi S.A. (2021). Nutraceutical Value of *Carica papaya*: A Review. Sci. Afr..

[5] Valencia Sandoval K., Duana Ávila D., Hernández Gracia T.J. (2017). Estudio Del Mercado de Papaya Mexicana: Un Análisis de Su Competitividad (2001–2015). Suma Negocios.

[6] Iturralde-García R.D., Cinco-Moroyoqui F.J., Martínez-Cruz O., Ruiz-Cruz S., Wong-Corral F.J., Borboa-Flores J., Cornejo-Ramírez Y.I., Bernal-Mercado A.T., Del-Toro-Sánchez C.L. (2022). Emerging Technologies for Prolonging Fresh-Cut Fruits’ Quality and Safety during Storage. Horticulturae.

[7] Ayón-Reyna L.E., Tamayo-Limón R., Cárdenas-Torres F., López-López M.E., López-Angulo G., López-Moreno H.S., López-Cervántes J., López-Valenzuela J.A., Vega-García M.O. (2015). Effectiveness of Hydrothermal-Calcium Chloride Treatment and Chitosan on Quality Retention and Microbial Growth during Storage of Fresh-Cut Papaya. J. Food Sci..

[8] González-Aguilar G.A., Valenzuela-Soto E., Lizardi-Mendoza J., Goycoolea F., Martínez-Téllez M.A., Villegas-Ochoa M.A., Monroy-García I.N., Ayala-Zavala J.F. (2009). Effect of Chitosan Coating in Preventing Deterioration and Preserving the Quality of Fresh-Cut Papaya “Maradol”. J. Sci. Food Agric..

[9] Kuwar U., Sharma S., Tadapaneni V.R.R. (2015). Aloe Vera Gel and Honey-Based Edible Coatings Combined with Chemical Dip as a Safe Means for Quality Maintenance and Shelf Life Extension of Fresh-Cut Papaya. J. Food Qual..

[10] Waghmare R.B., Annapure U.S. (2013). Combined Effect of Chemical Treatment and/or Modified Atmosphere Packaging (MAP) on Quality of Fresh-Cut Papaya. Postharvest Biol. Technol..

[11] Antunes M.D., Gago C.M., Cavaco A.M., Miguel M.G. (2012). Edible Coatings Enriched with Essential Oils and Their Compounds for Fresh and Fresh-Cut Fruit. Recent Patents Food Nutr. Agric..

[12] Fisher K., Phillips C.A. (2006). The Effect of Lemon, Orange and Bergamot Essential Oils and Their Components on the Survival of *Campylobacter jejuni*, *Escherichia coli* O157, *Listeria monocytogenes*, *Bacillus cereus* and *Staphylococcus aureus* In Vitro and in Food Systems. J. Appl. Microbiol..

[13] Ben Hsouna A., Ben Halima N., Smaoui S., Hamdi N. (2017). Citrus Lemon Essential Oil: Chemical Composition, Antioxidant and Antimicrobial Activities with Its Preservative Effect against Listeria Monocytogenes Inoculated in Minced Beef Meat. Lipids Health Dis..

[14] Himed L., Merniz S., Monteagudo-Olivan R., Barkat M., Coronas J. (2019). Antioxidant Activity of the Essential Oil of Citrus Limon before and after Its Encapsulation in Amorphous SiO_2_. Sci. Afr..

[15] Jyotirmayee B., Mahalik G. (2022). A Review on Selected Pharmacological Activities of *Curcuma longa* L.. Int. J. Food Prop..

[16] Ibáñez M.D., Blázquez M.A. (2021). *Curcuma longa* L. Rhizome Essential Oil from Extraction to Its Agri-Food Applications. A Review. Plants.

[17] Zou Y., Yu Y., Cheng L., Li L., Zou B., Wu J., Zhou W., Li J., Xu Y. (2021). Effects of Curcumin-Based Photodynamic Treatment on Quality Attributes of Fresh-Cut Pineapple. LWT.

[18] Chai Z., Zhang F., Liu B., Chen X., Meng X. (2021). Antibacterial Mechanism and Preservation Effect of Curcumin-Based Photodynamic Extends the Shelf Life of Fresh-Cut Pears. LWT.

[19] Tao R., Zhang F., Tang Q., Xu C.S., Ni Z.J., Meng X. (2019). Effects of Curcumin-Based Photodynamic Treatment on the Storage Quality of Fresh-Cut Apples. Food Chem..

[20] Lavinia M., Hibaturrahman S.N., Harinata H., Wardana A.A. (2020). Antimicrobial Activity and Application of Nanocomposite Coating from Chitosan and ZnO Nanoparticle to Inhibit Microbial Growth on Fresh-Cut Papaya. Food Res..

[21] Cortez-Vega W.R., Pizato S., De Souza J.T.A., Prentice C. (2014). Using Edible Coatings from Whitemouth Croaker (*Micropogonias furnieri*) Protein Isolate and Organo-Clay Nanocomposite for Improve the Conservation Properties of Fresh-Cut “Formosa” Papaya. Innov. Food Sci. Emerg. Technol..

[22] Tabassum N., Aftab R.A., Yousuf O., Ahmad S., Zaidi S. (2023). Application of Nanoemulsion Based Edible Coating on Fresh-Cut Papaya. J. Food Eng..

[23] Luciano W.A., Pimentel T.C., Bezerril F.F., Barão C.E., Marcolino V.A., de Siqueira Ferraz Carvalho R., dos Santos Lima M., Martín-Belloso O., Magnani M. (2023). Effect of Citral Nanoemulsion on the Inactivation of *Listeria monocytogenes* and Sensory Properties of Fresh-Cut Melon and Papaya during Storage. Int. J. Food Microbiol..

[24] Zielińska A., Carreiró F., Oliveira A.M., Neves A., Pires B., Venkatesh D.N., Durazzo A., Lucarini M., Eder P., Silva A.M. (2020). Polymeric Nanoparticles: Production, Characterization, Toxicology and Ecotoxicology. Molecules.

[25] Rahman N.A., Siddiquee S., Hong Melvin G.J., Rahman M.M. (2019). Applications of Polymeric Nanoparticles in Food Sector.

[26] Jafari S.M. (2017). An Overview of Nanoencapsulation Techniques and Their Classification.

[27] Ahmadi P., Jahanban-Esfahlan A., Ahmadi A., Tabibiazar M., Mohammadifar M. (2022). Development of Ethyl Cellulose-Based Formulations: A Perspective on the Novel Technical Methods. Food Rev. Int..

[28] Din M.I., Ghaffar T., Najeeb J., Hussain Z., Khalid R., Zahid H. (2020). Potential Perspectives of Biodegradable Plastics for Food Packaging Application-Review of Properties and Recent Developments. Food Addit. Contam. Part A Chem. Anal. Control Expo. Risk Assess..

[29] Galindo-Pérez M.J., Quintanar-Guerrero D., Cornejo-Villegas M.d.l.Á., Zambrano-Zaragoza M.d.l.L. (2018). Optimization of the Emulsification-Diffusion Method Using Ultrasound to Prepare Nanocapsules of Different Food-Core Oils. LWT.

[30] Xu D., Zhang J., Cao Y., Wang J., Xiao J. (2016). Influence of Microcrystalline Cellulose on the Microrheological Property and Freeze-Thaw Stability of Soybean Protein Hydrolysate Stabilized Curcumin Emulsion. LWT Food Sci. Technol..

[31] Wang Z.W., Duan H.W., Hu C.Y. (2009). Modelling the Respiration Rate of Guava (*Psidium guajava* L.) Fruit Using Enzyme Kinetics, Chemical Kinetics and Artificial Neural Network. Eur. Food Res. Technol..

[32] Iqbal T., Rodrigues F.A.S., Mahajan P.V., Kerry J.P., Gil L., Manso M.C., Cunha L.M. (2008). Effect of Minimal Processing Conditions on Respiration Rate of Carrots. J. Food Sci..

[33] Zambrano-Zaragoza M.d.l.L., Mercado-Silva E., Del Real L.A., Gutiérrez-Cortez E., Cornejo-Villegas M.A., Quintanar-Guerrero D. (2014). The Effect of Nano-Coatings with α-Tocopherol and Xanthan Gum on Shelf-Life and Browning Index of Fresh-Cut “Red Delicious” Apples. Innov. Food Sci. Emerg. Technol..

[34] Hagerman A.E., Austin P.J. (1986). Continuous Spectrophotometric Assay for Plant Pectin Methyl Esterase. J. Agric. Food Chem..

[35] Bradford M.M. (1976). A Rapid and Sensitive Method for the Quantitation of Microgram Quantities of Protein Utilizing the Principle of Protein-Dye Binding. Anal. Biochem..

[36] Waterhouse A.L. (2002). Determination of Total Phenolics. Curr. Protoc. Food Anal. Chem..

[37] Chien P.J., Sheu F., Yang F.H. (2007). Effects of Edible Chitosan Coating on Quality and Shelf Life of Sliced Mango Fruit. J. Food Eng..

[38] Sant’Anna V., Gurak P.D., Ferreira Marczak L.D., Tessaro I.C. (2013). Tracking Bioactive Compounds with Colour Changes in Foods—A Review. Dye. Pigment..

[39] Hasani S., Ojagh S.M., Ghorbani M. (2018). Nanoencapsulation of Lemon Essential Oil in Chitosan-Hicap System. Part 1: Study on Its Physical and Structural Characteristics. Biol. Macromol..

[40] Liu C., Liang B., Shi G., Li Z., Zheng X. (2015). Preparation and Characteristics of Nanocapsules Containing Essential Oil for Textile Application. Flavour Fragr. J..

[41] Pan K., Zhong Q., Baek S.J. (2013). Enhanced Dispersibility and Bioactivity of Curcumin by Encapsulation in Casein Nanocapsules. J. Agric. Food Chem..

[42] Zanotto-Filho A., Coradini K., Braganhol E., Schröder R., De Oliveira C.M., Simoes-Pires A., Battastini A.M.O., Pohlmann A.R., Guterres S.S., Forcelini C.M. (2013). Curcumin-Loaded Lipid-Core Nanocapsules as a Strategy to Improve Pharmacological Efficacy of Curcumin in Glioma Treatment. Eur. J. Pharm. Biopharm..

[43] Klippstein R., Wang J.T., El-gogary R.I., Bai J., Mustafa F., Rubio N., Bansal S., Al-jamal W.T. (2015). Passively Targeted Curcumin-Loaded PEGylated PLGA Nanocapsules for Colon Cancer Therapy In Vivo. Small.

[44] Umerska A., Gaucher C., Oyarzun-Ampuero F., Fries-Raeth I., Colin F., Villamizar-Sarmiento M.G., Maincent P., Sapin-Minet A. (2018). Polymeric Nanoparticles for Increasing Oral Bioavailability of Curcumin. Antioxidants.

[45] Suwannateep N., Banlunara W., Wanichwecharungruang S.P., Chiablaem K., Lirdprapamongkol K., Svasti J. (2011). Mucoadhesive Curcumin Nanospheres: Biological Activity, Adhesion to Stomach Mucosa and Release of Curcumin into the Circulation. J. Control. Release.

[46] Bagherifam S., Griffiths G.W., Mælandsmo G.M., Nyström B., Hasirci V., Hasirci N. (2015). Poly(Sebacic Anhydride) Nanocapsules as Carriers: Effects of Preparation Parameters on Properties and Release of Doxorubicin. J. Microencapsul..

[47] Galindo-Rodriguez S., Allémann E., Fessi H., Doelker E. (2004). Physicochemical Parameters Associated with Nanoparticle Formation in the Salting-out, Emulsification-Diffusion, and Nanoprecipitation Methods. Pharm. Res..

[48] Piirma I. (1992). Polymeric Surfactants.

[49] Sun C., Xu C., Mao L., Wang D., Yang J., Gao Y. (2017). Preparation, Characterization and Stability of Curcumin-Loaded Zein-Shellac Composite Colloidal Particles. Food Chem..

[50] Trujillo-Cayado L.A., Alfaro M.C., Muñoz J. (2018). Effects of Ethoxylated Fatty Acid Alkanolamide Concentration and Processing on D-Limonene Emulsions. Colloids Surf. A Physicochem. Eng. Asp..

[51] Pérez-Mosqueda L.M., Trujillo-Cayado L.A., Carrillo F., Ramírez P., Muñoz J. (2015). Formulation and Optimization by Experimental Design of Eco-Friendly Emulsions Based on d-Limonene. Colloids Surf. B Biointerfaces.

[52] Rao J., McClements D.J. (2012). Impact of Lemon Oil Composition on Formation and Stability of Model Food and Beverage Emulsions. Food Chem..

[53] Zhao S., Tian G., Zhao C., Li C., Bao Y., DiMarco-Crook C., Tang Z., Li C., Julian McClements D., Xiao H. (2018). The Stability of Three Different Citrus Oil-in-Water Emulsions Fabricated by Spontaneous Emulsification. Food Chem..

[54] Maringgal B., Hashim N., Mohamed Amin Tawakkal I.S., Muda Mohamed M.T. (2020). Recent Advance in Edible Coating and Its Effect on Fresh/Fresh-Cut Fruits Quality. Trends Food Sci. Technol..

[55] Hasan S.M.K., Ferrentino G., Scampicchio M. (2020). Nanoemulsion as Advanced Edible Coatings to Preserve the Quality of Fresh-Cut Fruits and Vegetables: A Review. Int. J. Food Sci. Technol..

[56] Wang D., Ma Q., Li D., Li W., Li L., Aalim H., Luo Z. (2021). Moderation of Respiratory Cascades and Energy Metabolism of Fresh-Cut Pear Fruit in Response to High CO_2_ Controlled Atmosphere. Postharvest Biol. Technol..

[57] Baldwin E.A., Bai J., Martín-Belloso O., Soliva-Fortuny R. (2011). Physiology of Fresh-Cut Fruits and Vegetables. Advances in Fresh-Cut Fruits and Vegetables Processing.

[58] Toivonen P.M.A., Brummell D.A. (2008). Biochemical Bases of Appearance and Texture Changes in Fresh-Cut Fruit and Vegetables. Postharvest Biol. Technol..

[59] Perdones A., Sánchez-González L., Chiralt A., Vargas M. (2012). Effect of Chitosan-Lemon Essential Oil Coatings on Storage-Keeping Quality of Strawberry. Postharvest Biol. Technol..

[60] Mohammadi A., Hashemi M., Hosseini S.M. (2015). Chitosan Nanoparticles Loaded with Cinnamomum Zeylanicum Essential Oil Enhance the Shelf Life of Cucumber during Cold Storage. Postharvest Biol. Technol..

[61] Zambrano-Zaragoza M.L., González-Reza R., Mendoza-Muñoz N., Miranda-Linares V., Bernal-Couoh T.F., Mendoza-Elvira S., Quintanar-Guerrero D. (2018). Nanosystems in Edible Coatings: A Novel Strategy for Food Preservation. Int. J. Mol. Sci..

[62] Ali A., Muhammad M.T.M., Sijam K., Siddiqui Y. (2011). Effect of Chitosan Coatings on the Physicochemical Characteristics of Eksotika II Papaya (*Carica Papaya* L.) Fruit during Cold Storage. Food Chem..

[63] Mochizuki M., Hirano M., Kanmuri Y., Kudo K., Tokiwa Y. (1995). Hydrolysis of Polycaprolactone Fibers by Lipase: Effects of Draw Ratio on Enzymatic Degradation. J. Appl. Polym. Sci..

[64] Perdones Á., Vargas M., Atarés L., Chiralt A. (2014). Physical, Antioxidant and Antimicrobial Properties of Chitosan-Cinnamon Leaf Oil Films as Affected by Oleic Acid. Food Hydrocoll..

[65] Malvano F., Corona O., Pham P.L., Cinquanta L., Pollon M., Bambina P., Farina V., Albanese D. (2022). Effect of Alginate-Based Coating Charged with Hydroxyapatite and Quercetin on Colour, Firmness, Sugars and Volatile Compounds of Fresh Cut Papaya during Cold Storage. Eur. Food Res. Technol..

[66] Zheng B., Zhang Z., Chen F., Luo X., McClements D.J. (2017). Impact of Delivery System Type on Curcumin Stability: Comparison of Curcumin Degradation in Aqueous Solutions, Emulsions, and Hydrogel Beads. Food Hydrocoll..

[67] Jayathunge K.G.L.R., Gunawardhana D.K.S.N., Illeperuma D.C.K., Chandrajith U.G., Thilakarathne B.M.K.S., Fernando M.D., Palipane K.B. (2014). Physico-Chemical and Sensory Quality of Fresh Cut Papaya (Carica Papaya) Packaged in Micro-Perforated Polyvinyl Chloride Containers. J. Food Sci. Technol..

[68] Price L.C., Buescher R.W. (2007). Decomposition of Turmeric Curcuminoids as Affected by Light, Solvent and Oxygen. J. Food Biochem..

[69] Schneider C., Gordon O.N., Edwards R.L., Luis P.B. (2015). Degradation of Curcumin: From Mechanism to Biological Implications. J. Agric. Food Chem..

[70] Ergun M., Huber D., Jeong J., Bartz J.A. (2006). Extended Shelf Life and Quality of Fresh-Cut Papaya Derived from Ripe Fruit Treated with the Ethylene Antagonist 1-Methylcyclopropene. J. Am. Soc. Hortic. Sci..

[71] Allanigue D.K.A., Sabularse V.C., Hernandez H.P., Serrano E.P. (2017). The Effect of Chitosan-Based Nanocomposite Coating on the Postharvest Life of Papaya (*Carica Papaya* L.) Fruits. Philipp. Agric. Sci..

[72] Velderrain-Rodríguez G.R., Ovando-Martínez M., Villegas-Ochoa M., Ayala-Zavala J.F., Wall-Medrano A., Álvarez-Parrilla E., Madera-Santana T.J., Astiazarán-García H., Tortoledo-Ortiz O., González-Aguilar G.A. (2015). Antioxidant Capacity and Bioaccessibility of Synergic Mango (Cv. Ataulfo) Peel Phenolic Compounds in Edible Coatings Applied to Fresh-Cut Papaya. Food Nutr. Sci..

[73] Karakurt Y., Huber D.J. (2003). Activities of Several Membrane and Cell-Wall Hydrolases, Ethylene Biosynthetic Enzymes, and Cell Wall Polyuronide Degradation during Low-Temperature Storage of Intact and Fresh-Cut Papaya (Carica Papaya) Fruit. Postharvest Biol. Technol..

[74] Formiga A.S., Pereira E.M., Junior J.S.P., Costa F.B., Mattiuz B.H. (2022). Effects of Edible Coatings on the Quality and Storage of Early Harvested Guava. Food Chem. Adv..

[75] Pilon L., Spricigo P.C., Miranda M., de Moura M.R., Assis O.B.G., Mattoso L.H.C., Ferreira M.D. (2015). Chitosan Nanoparticle Coatings Reduce Microbial Growth on Fresh-Cut Apples While Not Affecting Quality Attributes. Int. J. Food Sci. Technol..

[76] Galindo-Pérez M.J., Quintanar-Guerrero D., Mercado-Silva E., Real-Sandoval S.A., Zambrano-Zaragoza M.L. (2015). The Effects of Tocopherol Nanocapsules/Xanthan Gum Coatings on the Preservation of Fresh-Cut Apples: Evaluation of Phenol Metabolism. Food Bioprocess Technol..

[77] Macleod G.S., Fell J.T., Collett J.H. (1997). Studies on the Physical Properties of Mixed Pectin/Ethylcellulose Films Intended for Colonic Drug Delivery. Int. J. Pharm..

[78] Kagan V.E., Tyurina Y.Y. (1998). Recycling and Redox Cycling of Phenolic Antioxidants. Ann. N. Y. Acad. Sci..

[79] Zambrano-Zaragoza M.L., Gutiérrez-Cortez E., Del Real A., González-Reza R.M., Galindo-Pérez M.J., Quintanar-Guerrero D. (2014). Fresh-Cut Red Delicious Apples Coating Using Tocopherol/Mucilage Nanoemulsion: Effect of Coating on Polyphenol Oxidase and Pectin Methylesterase Activities. Food Res. Int..

